# Gait asymmetry in children with Duchenne muscular dystrophy: evaluated through kinematic synergies and muscle synergies of lower limbs

**DOI:** 10.1186/s12938-023-01134-7

**Published:** 2023-07-31

**Authors:** Qiliang Xiong, Yuan Liu, Jieyi Mo, Yuxia Chen, Lianghong Zhang, Zhongyan Xia, Chen Yi, Shaofeng Jiang, Nong Xiao

**Affiliations:** 1 Department of Biomedical Engineering, Nanchang Hangkong University, Nanchang, Jiangxi China; 2 Department of Rehabilitation, Children’s Hospital of Chongqing Medical University, Chongqing, China

## Abstract

**Background:**

Gait is a complex, whole-body movement that requires the coordinated action of multiple joints and muscles of our musculoskeletal system. In the context of Duchenne muscular dystrophy (DMD), a disease characterized by progressive muscle weakness and joint contractures, previous studies have generally assumed symmetrical behavior of the lower limbs during gait. However, such a symmetric gait pattern of DMD was controversial. One aspect of this is criticized, because most of these studies have primarily focused on univariate variables, rather than on the coordination of multiple body segments and even less investigate gait symmetry under a motor synergy of view.

**Methods:**

We investigated the gait pattern of 20 patients with DMD, compared to 18 typical developing children (TD) through 3D Gait Analysis. Kinematic and muscle synergies were extracted with principal component analysis (PCA) and non-negative matrix factorization (NNMF), respectively. The synergies extracted from the left and right sides were compared with each other to obtain a symmetry value. In addition, bilateral spatiotemporal variables of gait, such as stride length, percentage of stance and swing phase, step length, and percentage of double support phase, were used for calculating the symmetry index (SI) to evaluate gait symmetry as well.

**Results:**

Compared with the TD group, the DMD group walked with decreased gait velocity (both *p* < 0.01), stride length (both *p* < 0.01), and step length (both *p* < 0.001). No significant difference was found between groups in SI of all spatiotemporal parameters extracted between the left and right lower limbs. In addition, the DMD group exhibited lower kinematic synergy symmetry values compared to the TD group (*p* < 0.001), while no such significant group difference was observed in symmetry values of muscle synergy.

**Conclusions:**

The findings of this study suggest that DMD influences, to some extent, the symmetry of synergistic movement of multiple segments of lower limbs, and thus kinematic synergy appears capable of discriminating gait asymmetry in children with DMD when conventional spatiotemporal parameters are unchanged.

## Introduction

Duchenne muscular dystrophy (DMD) is a common muscular dystrophy in the pediatric population, characterized by progressive muscle weakness and joint contractures, which typically lead to loss of ambulation by the age of 13 years [1, 2]. Symmetrical behavior of the lower limbs during DMD gait has often been assumed, mainly for simplicity in data collection and analysis. Indeed, previous studies found that spatiotemporal parameters (i.e., stance time, swing time, step length, et al.) showed no significant difference between the left and right side in DMD’s gait [3, 4]. Consequently, data collected from the left and right sides during gait have typically been averaged [4], pooled together [3], or analyzed only on one side, such as the right side [5] or dominant leg [6]. However, such a symmetric gait pattern of DMD has been criticized as children with DMD may exhibit an asymmetric gait pattern, and as such, only selecting the gait features of the weakest side [7]. Furthermore, some other studies have reported little evidence of gait symmetry or asymmetry in individuals with DMD during gait analysis [8–10]. Within these studies, it is likely that there are inconclusive understanding of the occurrence of symmetric/asymmetrical gait patterns in individuals with DMD; a better understanding of the occurrence of asymmetrical gait patterns in individuals with DMD may lead to a more detailed understanding of the biomechanical factors that affect their gait ability and provide suggestions for more appropriate and effective rehabilitation and exercise prescription in clinical settings.

Therefore, this study was motivated by the controversial information about whether children with DMD change their gait symmetry, and one aspect where this is lacking is previous symmetry analysis only concerning the univariate variables, which may cause a form of bias, i.e., the inter-component covariance bias [11]. For example, lateral excessive trunk motion and contralaterally pelvic obliquity have been reported as a compensatory result for hip abductor muscle weakness during walking in children with DMD [12]. One way to consider such a compensatory strategy is using a model of motor synergy [13, 14]. Motor synergy is a hypothesis that the neuromotor system coordinates joint and muscle dimensionality in complex motor behaviors, with the central nervous system (CNS) simplifying control through a small number of functional units, or synergies [15–17]. This modular control hypothesis has been supported by the identification of motor synergies at different levels [15, 18], such as a few muscle synergies that can describe various activation patterns in human locomotion [19, 20], and a few kinematic synergies that can successfully explain joint angles in some typical human movements [21–23]. To sum up, the CNS organizes these redundant systems by generating the common motor command to coordinate multiple muscles or joints [24, 25]. This coordinated pattern of muscle contractions or joint angle movements can be extracted as common components using dimensionality reduction techniques, such as principal component analysis (PCA) [23, 26] or non-negative matrix factorization (NNMF) [27, 28].

Recent research has revealed that the core muscles of the body play an important role in scheduling, coordinating, and gathering strength for the limb regions before their actions [29, 30]. Clinically, due to muscle damage, the core muscles of children with DMD have remarkably reduced ability to control the limbs [31]. This also indicates that the movements among their limbs are not well-coordinated, and the movement between the left and right limbs may be gradually mismatched, leading to asymmetric compensatory gait. As a result, we wonder if differences in gait symmetry between DMD and TD children can be reflected from the perspective of motor synergy.

To test this hypothesis, we investigated the gait symmetry of 20 patients with DMD, compared to 18 typical developing children (TD) through 3D gait analysis and motor synergies approach. Kinematic synergies and muscle synergies were extracted with principal component analysis (PCA) and non-negative matrix factorization (NNMF), respectively. The synergies extracted from the left and right sides were compared with each other to obtain a symmetry value. As a comparative method, spatiotemporal gait parameters of each limb, such as stride length, percentage of the stance and swing phase, step length, and percentage of double support phase, were used for calculating the symmetry index (SI) to evaluate gait symmetry as well. Further details on the study procedures can be found in the following sections.

## Results

### Spatiotemporal parameters

**Fig. 1:**
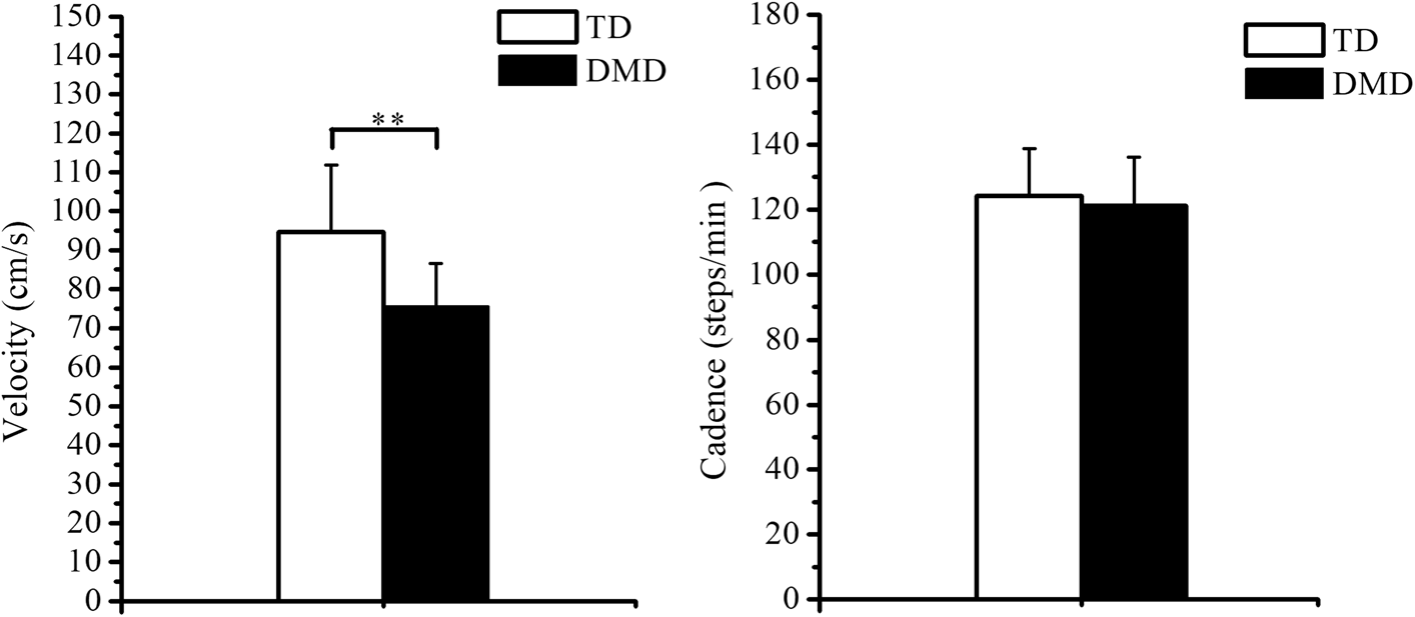
Group average values of walking velocity (left) and cadence (right) of the whole body for the TD and DMD group. ** indicates *p* < 0.01

Figure 1 shows the group average values of walking velocity and cadence of the whole body for the TD and DMD group, while Table 1 presents other bilateral spatiotemporal parameters of the left and right leg, including stride length, percentage of stance, percentage of swing, step length, and percentage of double support. The statistical analysis detected a significant difference between the two groups in terms of velocity, stride length, and step length. Furthermore, the DMD group exhibited reduced gait velocity (*p* < 0.01), stride length (*p* < 0.01), and step length (*p* < 0.01). Conversely, no significant difference was found in gait cadence (*p* > 0.05), percentage of stance (*p* > 0.05), percentage of swing (*p* > 0.05), and percentage of double support (*p* > 0.05).

**Table 1:** Bilateral spatiotemporal variables comparisons between the DMD and TD group

	Left side			Right side			SI of left and right side		
	TD	DMD	*p* value	TD	DMD	*p* value	TD	DMD	*p* value
Stride length (cm)	93.19 (17.36)	75.43 (11.33)	**< 0.01**	91.64 (17.05)	75.38 (11.27)	**< 0.01**	2.86 (1.97)	2.13 (2.46)	0.072
Stance (%)	59.28 (1.79)	59.66 (2.64)	0.718	61.28 (2.21)	60.47 (2.54)	0.377	3.59 (3.06)	3.81 (3.80)	0.851
Swing (%)	40.71 (1.79)	40.33 (2.64)	0.718^M^	38.71 (2.21)	39.52 (2.54)	0.377	5.59 (4.90)	5.65 (5.64)	0.784
Step Length (cm)	46.11 (8.57)	36.80 (6.04)	** < 0.01**	46.60 (9.42)	38.20 (5.79)	**< 0.01**	5.36 (7.47)	7.47 (5.74)	0.082
Double Support (%)	11.12 (2.73)	10.19 (2.36)	0.217	10.72 (1.77)	10.55 (2.34)	0.633	9.17 (10.23)	14.00 (9.89)	0.067

Data presented as mean (standard deviation)

The *p* values were calculated from Mann–Whitney *U* test

With regard to the spatiotemporal symmetry index (SI), the DMD group showed no significant difference compared to the TD group for all the five SI calculated from the bilateral spatiotemporal parameters (*p* > 0.05).

### Muscle synergies

**Fig. 2:**
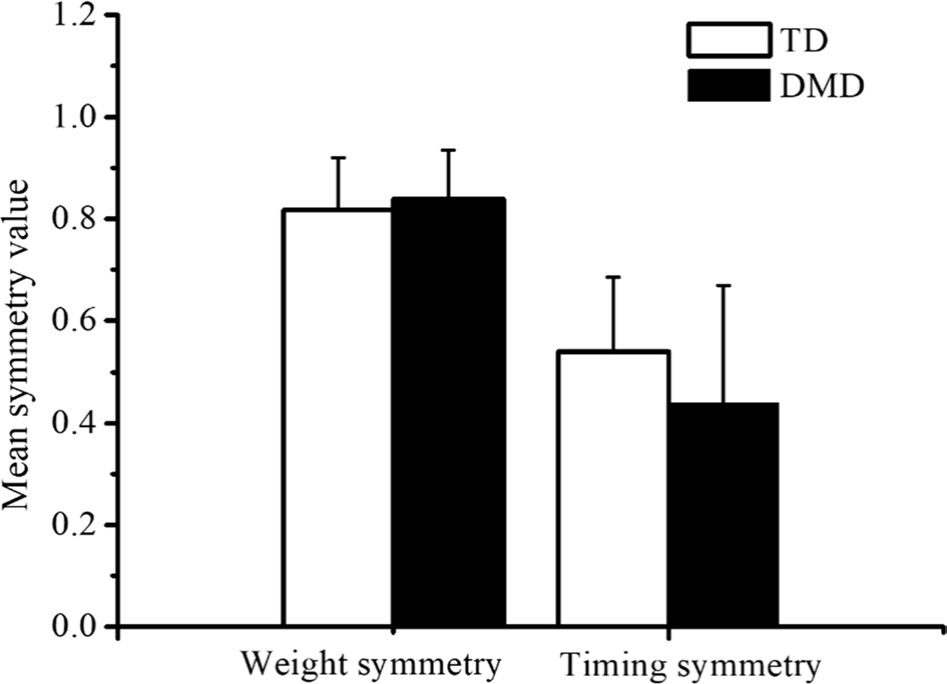
Mean symmetry value of muscle synergies for TD and DMD group. Left column: calculation according to the scalar dot product between the weight of muscle synergies on both sides. Right column: calculation according to the Pearson correlation coefficient between the corresponding timing coefficients of muscle synergies from both sides

Table 2 presents the number of muscle synergies identified in each subject, meeting the VAF threshold of > 90% overall and > 75% per muscle. All participants exhibited one to three muscle synergies, with 6 out of 18 TD subjects and 10 out of 20 DMD subjects showing no difference in the number of muscle synergies between the left and right side. Meanwhile, 11 out of 18 TD subjects and 8 out of 20 DMD subjects exhibited a left–right difference in the number of muscle synergies by one, and 1 out of 18 TD subjects and 2 out of 20 DMD subjects showed a left–right difference in the number of muscle synergies by two. In addition, no significant group difference was detected by statistical analysis concerning the mean weight symmetry value of muscle synergies (*p* = 0.553) or the mean timing symmetry value of muscle synergies (*p* = 0.133), as shown in Fig. 2.

**Table 2:** Number of kinematic synergies and muscle synergies identified in the TD and DMD group

	Kinematic synergy			Muscle synergy		
	Left	Right	Maximum of left and right	Left	Right	Maximum of left and right
TD	4.33 (0.59)	4.33 (0.59)	4.50 (0.51)	1.94 (0.63)	2.00 (0.76)	2.33 (0.59)
DMD	4.05 (0.39)	3.85 (0.58)	4.15 (0.48)	2.00 (0.64)	2.00 (0.56)	2.30 (0.57)

### Kinematic synergies

According to the variance threshold (> 90% explained variance) defined by previous studies [32], the study identified three to five kinematic synergies for each side across all participants, with 12 out of 18 subjects in the TD group and 12 out of 20 subjects in the DMD group displaying no difference in the number of kinematic synergies between the left and right side. Moreover, 6 out of 18 subjects in the TD group and 8 out of 20 subjects in the DMD group identified a left–right difference in the number of kinematic synergies by only one. The statistical analysis revealed that there was no significant difference between the TD and DMD groups in terms of the number of kinematic synergies extracted for both the left and right sides. Table 2 provides the number of kinematic synergies for each side and the corresponding maximum number of values.

**Fig. 3:**
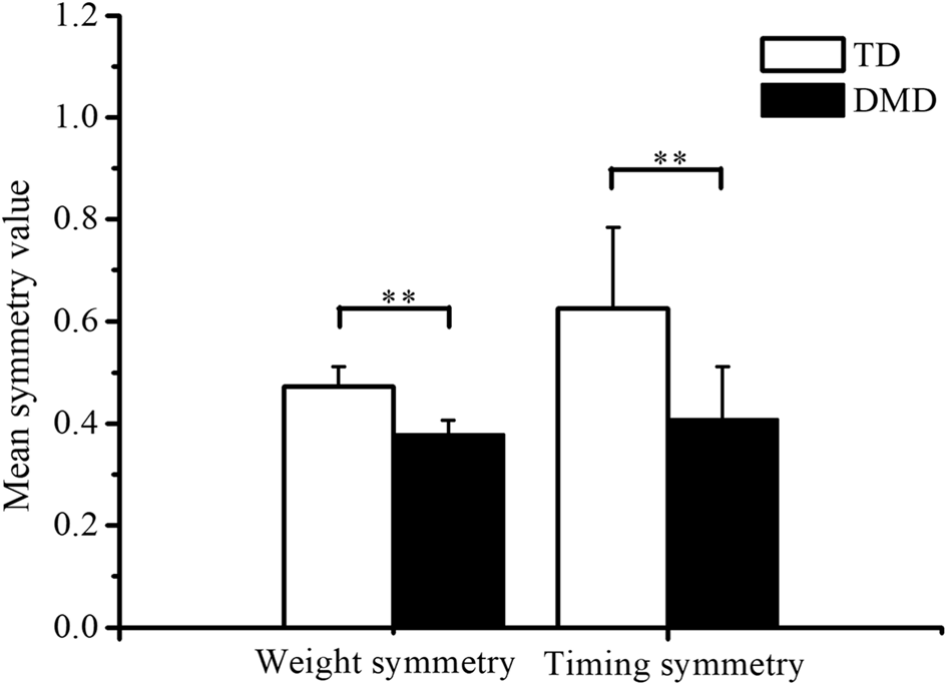
Mean symmetry value of kinematic synergies for TD and DMD group. The left column shows the values based on the scalar dot product between the weight of kinematic synergies on both sides, while the right column shows the values based on the Pearson correlation coefficient between the corresponding timing coefficients of kinematic synergies on both sides. ** indicates *p* < 0.01

The statistical analysis with the weight symmetry and timing symmetry value of kinematic synergy both detected a significant group difference between TD and DMD. As shown in Fig. 3, children in the DMD group exhibited a lower mean weight symmetry value (*p* < 0.01) and timing symmetry value (*p* < 0.01), compared with the TD group.

## Discussion

The primary objective of this study was to examine differences in gait symmetry patterns in individuals with DMD, with a particular focus on multiple joint and muscle synergies compared to healthy controls. The DMD group exhibited lower kinematic synergy symmetry values compared to the TD group, while no significant group difference was found in the conventional spatiotemporal symmetry index (SI). On the bases of the results obtained, the symmetry analysis throughout synergies extraction of multiple body segments seems to represent a valid measure of gait asymmetry in individuals with DMD.

### The symmetry of spatiotemporal parameters

The results obtained from spatiotemporal parameters in the current study agree with previous DMD’s gait studies, which confirmed that gait velocity showed a tendency to a reduction, without a statistical significance in [3, 33], but with statistical significance in our study. This discrepancy could be due to the normalization of gait velocity. For non-normalized self-selected walking speed, all studies agreed that children with DMD walked slower than TD children, while this difference disappeared when walking speed was normalized to height in [3]. In addition, a decrease in stride and step length was reported in the group of DMD, which was consistent with previous studies [5, 8, 10, 34]. While in the current study, there was no difference between the groups in terms of demographic information, compared with the control group, the DMD group had an increased extent of the area with sensory loss and severity of DMD, decreased ankle joint range of motions, and weaker foot muscle strength [35, 36]; therefore, these may be interpreted as a preparative factor for the alterations in spatiotemporal gait parameters.

The results of the statistical analysis showed no significant group differences (*p* > 0.05) for the five symmetry indices of gait stride length, swing time, stance time, step length, and double stance time. To our knowledge, no symmetry indices of gait spatiotemporal performances exist for this specific population. However, such symmetric gait spatiotemporal in individuals with DMD has been examined by comparing the spatiotemporal variables of both sides. For example, Maria Grazia D’Angelo et al. [3] investigated the gait pattern of 21 patients with DMD, compared to 10 healthy controls through 3D gait analysis. An initial comparison between the right and left limbs was made before the comparison with a control group and no statistical difference in gait parameters was found between the two limbs. Combined with our results, it is suggested that DMD has limited influence on the spatiotemporal symmetry of gait.

### The symmetry of muscle synergies

In addition to spatiotemporal parameters, we tried to verify the effect of DMD disease on the gait symmetry obtained through two levels of motor synergies: muscle synergy and kinematic synergy. According to the conceptual model of motor synergy, it has been assumed that the joints or muscles are engaged in groups with a fixed weight, i.e., the weight of synergies, to overcome the complexity of controlling a large number of degrees of freedom (DoF) [36, 37]. Based on this hypothesis, the CNS generates a large range of physical activities by the flexible recruitment of a limited number of synergies over time, i.e., the timing coefficients of synergies. That is, the symmetry of synergy weight and timing coefficients can be interpreted as the marker of CNS status [27, 28]. Looking at our results, the DMD group exhibited no significant alterations of muscle synergy symmetry values of weight and timing coefficient, compared to the TD group (*p* > 0.05), the absence of significant results is consistent with previous studies, suggesting DMD has little effect on the muscle synergies of lower limbs during gait. As we mentioned before, the most prominent symptom in DMD is muscle weakness, which can be the result of altered motor commands due to (1) lesions in the CNS [37], (2) disruptions in signal transmission between the CNS and the muscles [38], and/or (3) changes in the muscle itself [39]. In children with DMD, the main cause of muscle weakness appears to be a change in muscle structure[40]. That is, there is no evidence to support that muscle weakness in DMD has a neurological cause [41]. Taking together, the findings of the lack of significant difference concerning the symmetry of muscle synergies are in line with previous research, suggesting non-neural alterations have limited influence on muscle synergies of gait.

### The asymmetry of kinematic synergies

Considering the systematic relations between kinematic synergies and muscle synergies, it was postulated that muscle synergies are the source of kinematic synergies [42, 43], and the symmetry of kinematics synergies should be able to reveal similar age-related changes as muscle synergies. However, our results demonstrated that the DMD group exhibited significantly lower symmetry values of kinematic synergy weight and timing coefficient compared to the TD group (*p* < 0.001), while no significant group difference was observed in symmetry values of muscle synergy (*p* > 0.05). It appears that the effect of DMD is only manifested in the kinematic synergies and not in the muscle synergies. However, as previously discussed, DMD is a prevalent disorder of muscular dystrophy caused by mutations in the dystrophin gene, rather than neural damage. This may raise questions about how to interpret the asymmetry of kinematic synergy symmetry if it is not due to neural factors?

The observed asymmetries of kinematic synergies may be influenced by several factors. For example, the main role of the CNS in generating kinematic synergy has been questioned by other authors who suggested that the CNS may not directly control the kinematics (joint angles), but the “joint constraint” [44], which could potentially interfere with the coordinative structure required for optimizing postural stability. In addition, joint contractures represent a prevalent issue for the locomotor system of children with DMD and the passive moments produced by such contractures may have a positive effect on gait in individuals with muscle weakness [41]. Considering this case, it may be argued that the asymmetric kinematic synergy observed in the DMD group could be attributed to an unbalanced postural capacity to modulate multiple joint constraints of each limb during gait. The modifications in gait observed in the DMD group may be interpreted as strategies employed to cope with possible joint contractures and to provide support, propulsion, and balance during gait. Furthermore, it has been observed that young children with DMD adopt subtle compensatory strategies to reduce the demand on their weak muscles during gait, these strategies become more pronounced and unstable with increasing age [10, 45]. Combined with the lack of significant findings about symmetric spatiotemporal variables, it is possible that the progressive joint contractures observed in children with DMD may not affect both sides symmetrically. Instead, it is plausible that each joint segment may compensate separately for the disease deficit to preserve a global spatiotemporal invariance [46].

### Clinical implications and limitations

Many pieces of clinical evidence have indicated that compensatory gait is closely related to the progression of DMD [47], and quantitative evaluation of gait asymmetry in patients with DMD can reflect the condition of DMD [45]. However, as we mentioned in the introduction section, extracting kinematic parameters from the individual body segments hardly reflects the compensatory movements of multiple body segments. For example, it has previously been suggested that the lower power generation at the hip could be explained by weakness of the hip extensors, resulting in a pelvic anterior tilt, and consequently a more flexed position of the hip as a compensation mechanism [8, 48]. In the current study, we used the kinematic synergies to cluster the hip, knee, ankle, trunk, and pelvis variables as several synergies. The number of detected synergies did not differ significantly between the groups (see Table 2), while a higher number of synergies was believed to reflect a better neural control of the limb [27]. As such, our results suggest that the left and right limbs of children with DMD possess normative control commands for kinematic synergies (the same number of synergies), and any asymmetrical joint coordination (weight and timing structure of synergies) is largely attributed to compensatory strategies used to manage potential unbalanced non-neural alterations, such as weakness of the hip extensors. Based on the results of the present study, we propose that a reasonable rehabilitation objective would be to increase gait symmetry during DMD’s gait, using appropriate and effective exercise to overcome or correct the bilateral compensatory motions of the pelvis and lower extremities.

Although the promise of measuring gait symmetry and the underlying compensatory mechanism that contributes to gait asymmetry in children with DMD is exciting. We acknowledge some limitations in this study. One potential limitation is the limited sample size. Accordingly, we still use the Mann–Whitney *U* test to compare group differences, despite some variables having a normal distribution. Second, due to the limited sample size, the current study did not screen DMD patients according to the severity of the disease. Future studies should separately study patients with different disease courses, which would have better clinical significance for follow-up management and the development of an evaluation system for DMD patients.

## Conclusion

In conclusion, patients with DMD may present an asymmetric gait pattern, which is not manifested in individual variables of lower limbs, such as spatiotemporal parameters. However, if the kinematic variables are studied globally, an asymmetric gait pattern emerges. Furthermore, using such a model of kinematic synergies appears capable of discriminating gait asymmetry due to the compensatory strategies in children with DMD, and be helpful for a more appropriate and effective rehabilitation and exercise prescription.

## Methods

### Participants

20 male individuals with DMD, aged between 4 and 12 years, were included in the study. The inclusion criteria were: (1) confirmed DMD diagnosis (by clinical history, genetic testing, and muscle biopsy), (2) independent walking, and (3) no difficulty understanding the instructions. Exclusion criteria consisted of the presence of other disorders and previous surgical procedures. Individuals with other disorders and previous surgical procedures were excluded from the study. In addition, 18 developmental-age-matched healthy children were recruited as “typical development (TD)” controls. Table 3 shows a comprehensive overview of the participants' demographic and anthropometric characteristics. Before the study, all participants were required to provide informed consent, either personally or through parental consent. The study was approved by the ethics committee of the Children’s Hospital of Chongqing medical university.

**Table 3:** Participants' demographic and anthropometric characteristics

	TD	DMD
Participants #	18	20
Age (years)	7.55 ± 2.52	7.25 ± 2.33
Height (cm)	125.29 ± 17.92	119.05 ± 11.39
Weight (kg)	26.80 ± 8.74	23.90 ± 9.12
Body Mass index (kg m^−2^)	16.70 ± 2.34	16.38 ± 3.29

Values are expressed as mean ± SD

### Data acquisition

**Fig. 4:**
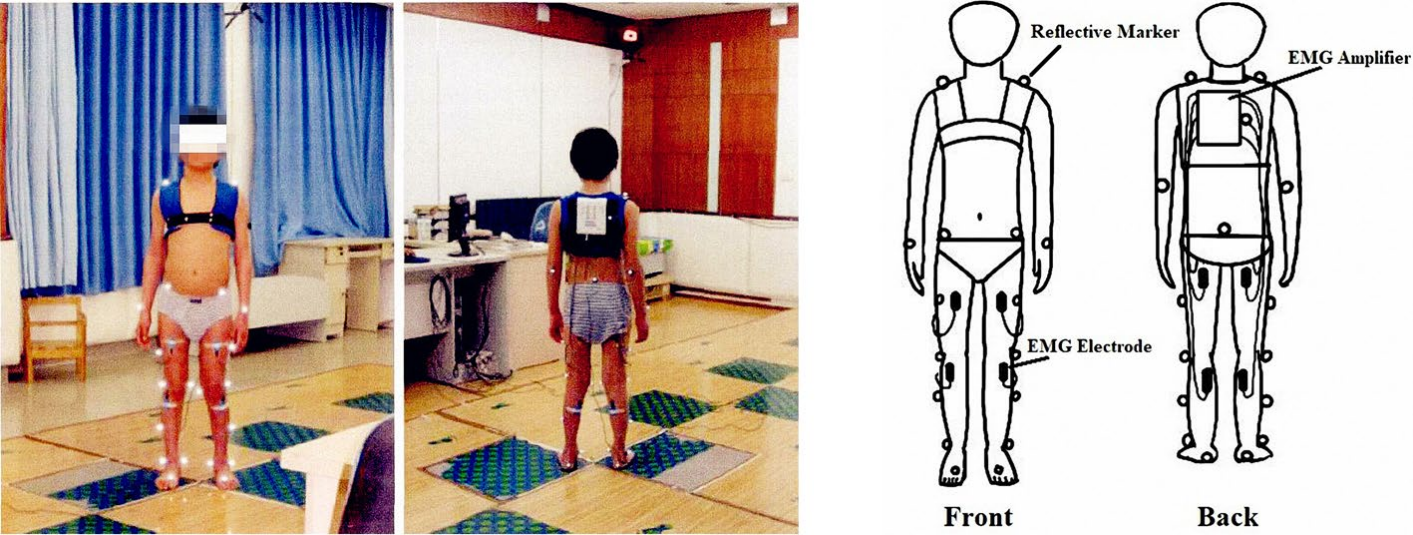
Schematic overview of the data collection protocol. The participants underwent a 3DGA model with total-body kinematics

The study utilized six high-speed digital cameras to record kinematic data of the participants at a sampling rate of 60 frames per second, via a motion capture system (Raptor-E, Motion Analysis, Santa Rosa, CA, USA). According to the guideline of Helen Hayes (HH) set [49], the participants' shoulder (lateral to the acromion), elbow (lateral epicondyle), wrist (ulnar styloid process), hip (posterior superior iliac spine), knee (lateral joint line), ankle (lateral malleolus), and trunk (shoulder blade) were affixed with reflective markers, as depicted in Fig. 4. Multiple trials were conducted with participants walking barefoot at their preferred pace along a 7-m path containing force plates, enabling the determination of individual strides with full marker visibility. Furthermore, surface EMG was recorded from the quadriceps femoris, hamstrings, tibialis anterior, gastrocnemius, and gluteus maximus. The MA-300 EMG system (Motion Lab Systems, Inc, USA), recorded EMG data at a rate of 2000 Hz.

The study defined a successful sequence of alternate steps as one that consisted of at least three complete, consecutive strides. From each participant, ten such trials were analyzed. Only walking sequences in which the participant walked straight without stopping or diverting were selected for data analysis. In addition, to mitigate the effect of walking acceleration or deceleration, the first and last steps of each sequence (beginning and stopping) were excluded from the data analysis. The EMG recording, marker tracking, and ground response forces were all synchronized.

### Data analysis

#### Data pre-processing

The raw kinematic data were initially smoothed using a Butterworth 2nd order low pass filter with a 10 Hz cutoff frequency. The joint angles of the bilateral lower extremities were then calculated using OrthoTrak (Motion Analysis, USA). OrthoTrak is a three-dimensional orthopedic gait analysis software, which can accept 3D coordinates from a motion capture system and output the kinematics measures, such as spatiotemporal parameters, joint angles, trunk forward/lateral tilt angle et al. In the current study, the walking velocity and cadence values of the whole body were calculated using the 3D trajectory of the V. sacral marker, which is located at the sacral area. Other bilateral spatiotemporal parameters such as stride length, percentage of stance phase time, percentage of swing phase time, step length, and percentage of double support were accordingly calculated from the 3D trajectory of the left and right heel marker. The joint angles for each gait stride were segmented based on the timing of the heel strike. Specifically, the point, where the elevation of the heel reflective marker was at the lowest point was determined as the heel strike. To account for differences in stride duration between trials and subjects, all strides were interpolated as a percentage of gait stride time (0–100%). Each participant's normalized trials for each joint were averaged across trials. Then, the averaged joint angles were normalized to have zero mean and unit variance.

#### Kinematic synergies extraction

A multi-joint synergy model was created for each lower limb during walking, encompassing 15 degrees of freedom (DoFs). The model included 3 DoFs each for the hip (flexion–extension (Hip-Flex), abduction–adduction (Hip-Abd), and internal–external rotation (Hip-Rot)), knee (flexion–extension (Knee-Flex), abduction–adduction (Knee-Abd), and internal–external rotation (Knee-Rot)), and ankle (flexion–extension (Ankle-Flex), abduction–adduction (Ankle-Abd), and internal–external rotation (Ankle-Rot)). In addition, 3 DoFs of trunk movements including lateral tilt (Trunk_Lat_Tilt), forward tilt (Trunk_Fwd_Tilt) and rotation (Trunk_Rotation), and 3 DoFs of pelvis movements including lateral tilt (Pelvis_Lat_Tilt), forward tilt (Pelvis _Fwd_Tilt) and rotation (Pelvis _Rotation) were also included for the following PCA processing.

The kinematic data for the 15 DoFs were first consolidated into an original motion matrix (${M}^{m\times t}$) for each participant, allowing for the extraction of kinematic synergies for each side. The original joint motion matrix was then decomposed into two components: synergy weight (*W*, or synergy structure), and timing coefficient (*C*, or relative time-varying activation of those synergies), as the equation below.

$${M}^{m\times t}\cong {W}^{m\times n}{C}^{n\times t}$$

The number of principal components (PCs) or synergies, denoted by *n*, was used in conjunction with the number of degrees of freedom (DoFs), denoted by m, to compute the timing coefficient, also referred to as *C*. The timing coefficient matrix has dimensions of ${n \times t}$, with t representing the number of timepoints (101 in this study) across the normalized gait cycle. The number of synergies is determined by the number of PCs, which corresponds to the degree of variance explained, with the first PCs accounting for the highest variance, and so on [50]. Following previous research criteria, synergies were considered plausible if they explained more than 90% of the variance [32, 51].

#### Muscle synergies extraction

To eliminate the influence of power interference, the surface electromyography (sEMG) signals underwent band-pass filtering with a fourth-order zero-phase Butterworth digital filter between 10 and 400 Hz, and a 50 Hz digital notch filter was applied. The EMG signals were segmented into gait strides based on the onset of heel contact. To derive the linear envelope, the pre-processed EMG signals were demeaned, rectified, and subjected to low-pass filtering using a zero-lag fourth-order low-pass filter with a 9 Hz cutoff frequency [52, 53]. The envelope of each muscle was then normalized to its highest value across all trials, resampled at 1% intervals over the 0–100% stride duration, and averaged across all strides for each subject, generating a 5 (muscles) × 101 (0–100% stride duration) matrix for each lower limb [54].

To extract muscle synergies from the EMG data, the non-negative matrix factorization (NNMF) technique was utilized, which satisfies the physiological relevance of muscle activation [27, 28, 55]. The measured EMG data matrices (*V*) were decomposed into two components, spatial structure (*S*) and timing coefficient (*H*), using the following equation:

$${ V}^{m\times t}\cong {S}^{m\times n}{H}^{n\times t}$$

In this equation, *S* is an $m\times s$ matrix, where m is the number of muscles (in this study *m* = 5) and *n* is the number of muscle synergies. *H* is an $n\times t$ matrix, where t is the number of timepoints (101 across the normalized gait cycle in this study). Thus, each column of *S* represents the relative weight of muscles in each synergy and each row of *H* represents the activation level of each synergy over the gait cycle. Non-negative matrix factorization (NNMF) was employed with an iterative optimization approach to minimize the error between the calculated activations from the product of *S* and *H* (*S* × *H*) and the measured EMG data matrices (*V*) [56].

Regarding the number of muscle synergies needed for accurate reconstruction of the original EMG data matrices (*V*), no assumptions were made in this study. The variance accounted for (VAF, ranging from 0 to 1), which is defined as $\mathrm{VAF}=1-{\Vert \varepsilon \Vert }^{2}/{\Vert M\Vert }^{2}$ [27, 28, 57], was used to measure the goodness of fit of the data reconstruction. For each subject, we identified the least number of muscle synergies that satisfied the following 2 criteria: (1) the total reconstructed EMGs counted for at least 90% of the variance across all muscles (VAF > 90%); (2) each reconstructed EMGs counted for greater than 75% VAF of the measurement from the corresponding single muscle. These criteria are thought to be conservative to guarantee the accuracy of the reconstruction [28].

#### Symmetry values calculation based on the kinematic and muscle synergies

It is important to note that a direct comparison between the left and right sides can be facilitated by imposing the same number of kinematic synergies or muscle synergies on both sides. In previous studies, the number of kinematic or muscle synergies has been determined based on a variance threshold value of 90%. However, this approach can result in the left and right sides having different numbers of synergies, which can complicate direct comparisons between them. To overcome this limitation, we selected the maximum number of synergies on the left and right sides as the number of synergies used for symmetry comparison. For instance, if three synergies were identified for the left and four for the right side according to the variance threshold, the number of synergies was set to four for both sides. Then, the scalar dot product was used to compare the weight of kinematic or muscle synergies on the left to the right side. The pair with the highest value was selected, and the process was repeated until all kinematic or muscle synergies were matched. This matching process was repeated for all subjects.

**Fig. 5:**
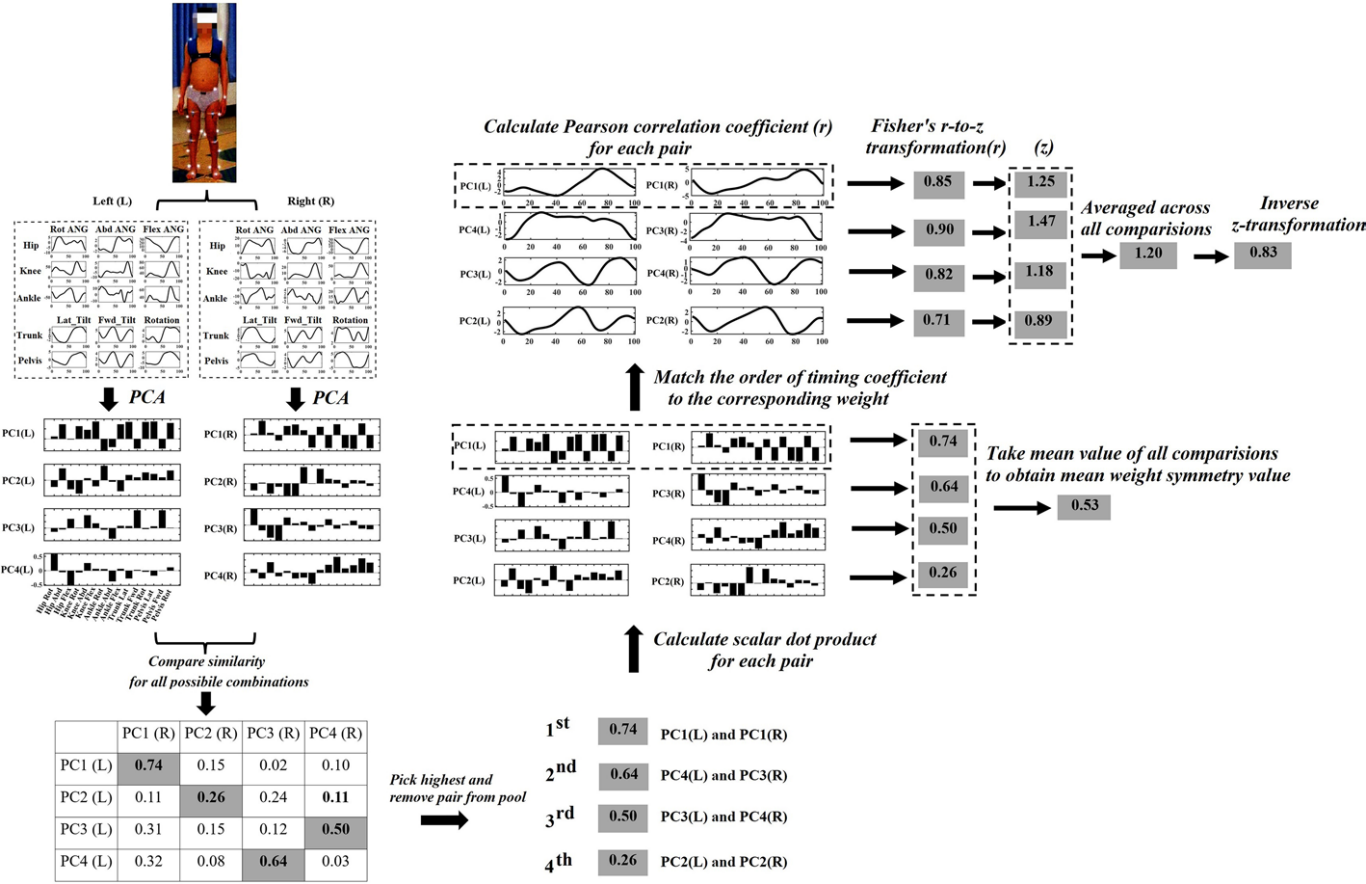
Schematic overview of the calculation steps for the mean symmetry values of kinematic synergies. In Step 1, joint angles of the hip, knee, and ankle concerning flexion–extension, abduction–adduction, internal–external rotation, and trunk and pelvis motions including lateral tilt, forward tilt, and rotation angles were derived from the 3DGA model. These 15 independent variables were presented during one gait cycle for each side, which were then pooled together to form an original matrix. In Step 2, Principal Component Analysis (PCA) was utilized to decompose the original matrix into two components, namely, synergy weight and timing coefficient. In Step 3, the scalar dot product was employed to compute the similarities between all possible combinations of the synergy weight on the left and right sides. The highest value pair was then eliminated from the pool, and the process was repeated until all kinematic synergies were matched. Step 4 involved the comparison of the weight of synergies on both sides of each pair with the scalar dot product. The mean weight symmetry value was determined by averaging the symmetry value of all comparisons. In Step 5, the Pearson correlation coefficient (*r*) was utilized to assess the similarity of the corresponding timing coefficients for each pair. Fisher’s *r*-to-*z* transformation was employed to normalize the *r* values, which were then averaged across all comparisons. The inverse *z*-transformation was applied to transform the averaged *z* value back to *r*, representing the mean timing symmetry value

Following the matching process, a comparison between the synergy weights (${W}^{m\times n}$) and their corresponding timing coefficients (${C}^{n\times t}$) of the left and right sides were conducted to assess their symmetry. The weight symmetry and timing symmetry values were then derived as the measures of symmetry for the respective synergy weight and timing coefficient. To calculate the weight symmetry, the scalar dot product was used to compare the weight of synergies for each pair. The similarity of the corresponding timing coefficients for each pair was assessed using the Pearson correlation coefficient (r). To account for intraindividual variance, these r values were normalized using Fisher's r-to-z transformation and then averaged across all comparisons. Finally, the inverse z-transformation was performed to obtain the mean timing symmetry value. An infographic demonstrating the synergies matching and symmetry value calculation process is depicted in Fig. 5.

#### The spatiotemporal symmetry index calculation

As noted by Viteckova et al. [58], the symmetry index (SI) is the most frequently used and cited method in studies investigating gait symmetry. In line with this approach, basic bilateral spatiotemporal parameters of gait, including stride length, percentage of stance phase time, percentage of swing phase time, and percentage of double support, were obtained from each walking trial for comparative analysis. Specifically, the average values of these spatiotemporal parameters were computed for each participant across all trials and subsequently utilized to compute the SI using the following formula.

$$\text{SI}=\frac{(\left| {{X}_{L}}-{{X}_{R}} \right|)}{0.5\cdot ({{X}_{L}}+{{X}_{R}})}\cdot 100 $$

where ${X}_{L}$ and ${X}_{R}$ are spatiotemporal variables of the left and right side, respectively. The value of SI = 0 indicates full symmetry [59].

### Statistical analysis

The normality of the data was assessed by performing the Shapiro–Wilk test. The results showed that the demographic characteristics, weight symmetry of kinematic synergies, and weight and timing symmetry of muscle synergies were normally distributed. However, other variables did not meet the normality assumption. Combined with the limitation of sample size, the Mann–Whitney U test was used to compare these variables between groups (DMD vs. TD). All statistical analyses were conducted using IBM SPSS software (IBM, Armonk, NY, USA). For all statistical tests, results were considered statistically significant when *p* < 0.05.

## Data Availability

The data sets generated and/or analyzed during the current study are not publicly available due to clinical policy but are available from the corresponding author on reasonable request.
